# Supplementary material for: Hypoxia-driven deSUMOylation of EXOSC10 promotes adaptive changes in the transcriptome profile
Source: Cell Mol Life Sci. 2024 Jan 27;81(1):58. doi: 10.1007/s00018-023-05035-9 (PMC10817850; doi:10.1007/s00018-023-05035-9)
Supplement: Supplementary file 1 — Supplementary file1 (PDF 4267 KB) [file 18_2023_5035_MOESM1_ESM.pdf]

Supplementary information

Title: Hypoxia-driven deSUMOylation of EXOSC10 promotes adaptive changes in the transcriptome profile

Chrysa Filippopoulou[1], Chairini C. Thomé[2], Sofia Perdikari[3], Evgenia Ntini[3], George Simos[1,4], Katherine E. Bohnsack[2] and Georgia Chachami[1,*]
, *
[1]*Laboratory of Biochemistry, Faculty of Medicine, University of Thessaly, Biopolis 41500, Larissa, Greece*
[2] *Department of Molecular Biology, University Medical Center Göttingen, 37073, Germany*
[3] *Institute of Molecular Biology and Biotechnology, Foundation for Research and Technology-Hellas (FORTH), Heraklion 70013, Greece.*
[4]*Gerald Bronfman Department of Oncology, Faculty of Medicine, McGill University, Montreal, Canada*

***To whom correspondence should be addressed: ghah@med.uth.gr**

Supplementary Table S1: List of the DNA oligonucleotides used in this study.

oligonucleotides	Sequence	Application	Reference
EXOSC10 For BamHI	5’-TCAGGATCCATGGCGCCAC-3’	Molecular cloning pcDNA 3.1-HA-EXOSC10/ pBluescript II SK (+/-)-EXOSC10	Sigma-Aldrich, StLouis, MO, USA
EXOSC10 Rev XbaI	5’-CTCTAGATCATCTCTGTGGCC-3’	Molecular cloning pcDNA 3.1-HA-EXOSC10/ pBluescript II SK (+/-)-EXOSC10	Sigma-Aldrich, StLouis, MO, USA
EXOSC10 K583R mutag.	5’-AGATGCCCCTGCTCAGGTCTGAAGTTGCAG-3’	Site-directed mutagenesis ($EXOSC10_{K583R}$)	Eurofins
EXOSC10 K583R compl. mutag.	5’-CTGCAACTTCAGACCTGAGCAGGGGCATCT -3’	Site-directed mutagenesis ($EXOSC10_{K583R}$)	Eurofins
EXOSC10 K168R mutag.	5’TGGCAAAAAAGCAAGATCTGAAACTTTCCG-3’	Site-directed mutagenesis ($EXOSC10_{K168R}$)	Eurofins
EXOSC10 K168R compl mutag.	5’-CGGAAAGTTTCAGATCTTGCTTTTTTGCCA-3’	Site-directed mutagenesis ($EXOSC10_{K168R}$)	Eurofins
EXOSC10 K201R mutag.	5’-CACCATTTCTTCCTAGAATCTTCATCAAACC-3’	Site-directed mutagenesis ($EXOSC10_{K201R}$)	Eurofins
EXOSC10 K201R compl mutag.	5’-GGTTTGATGAAGATTCTAGGAAGAAATGGTG-3’	Site-directed mutagenesis ($EXOSC10_{K201R}$)	Eurofins
EXOSC10 *D313A mutag.*	5’-GAATTGTCAGGAATTTGCAGTTAACTTGGAGCACCACTCTTACAGG-3’	Site-directed mutagenesis ($EXOSC10_{D313A}$)	Eurofins
EXOSC10 D313A *compl mutag.*	5’-CCTGTAAGAGTGGTGCTCCAAGTTAACTGCAAATTCTGACAATTC-3’	Site-directed mutagenesis ($EXOSC10_{D313A}$)	Eurofins

3’18S – 5’ITS1	5’-CCTCGCCCTCCGGGCTCCGTTAATGATC-3’	Northern blot probe	[1]
ITS2	5’-GCTCTCTCTTTCCCTCTCCGTCTTCC-3’	Northern blot probe	Eurofins
3’5.8S – 5’ITS2	5’GGGGCGATTGATCGGCAAGCGACGCTC-3’	Northern blot probe	[2]
16S	5’-TTCTATAGGGTGATAGATTGGTCC-3’	Northern blot probe	Eurofins
mt-tRNA^{Phe}	5’-TTGCTTTGAGGAGGTAAGCTACATAA-3’	Northern blot probe	Eurofins
28S	5’- TACCTCTTAACGGTTTCACGCCCTCTTGAACTCTC-3’	Northern blot probe	Eurofins
5S	5’-GAGATCAGACGAGATCGGGCGCGTTC-3’	Northern blot probe	Eurofins

Supplementary Table S2: List of plasmids used in this study.

Plasmid	Application
pcDNA 3.1-HA-EXOSC10 WT	Overexpression in human cell lines
pcDNA 3.1-HA-EXOSC10 K583R	Overexpression in human cell lines
pcDNA 3.1-HA-EXOSC10 K168R	Overexpression in human cell lines
pcDNA 3.1-HA-EXOSC10 K201R	Overexpression in human cell lines
pcDNA5-2xFlag-His6-siinsEXOSC10 WT	Generation of stable HeLa FlpIn cell lines
pcDNA5-2xFlag-His6-siinsEXOSC10 K583R	Generation of stable HeLa FlpIn cell lines
pGEX-4T1-EXOSC10 WT	Protein expression in *E.coli*
pGEX-4T1-EXOSC10 K583R	Protein expression in *E.coli*
pGEX-4T1-EXOSC10 K168R	Protein expression in *E.coli*
pGEX-4T1-EXOSC10 K201R	Protein expression in *E.coli*
pGEX-4T1-EXOSC10 D313A	Protein expression in *E.coli*
pGEX-4T1-USP36 1-420	Protein expression in *E.coli* (a kind offer from Dr. M.Dai)
pGEX-4T1-USP36 421-800	Protein expression in *E.coli* (a kind offer from Dr. M.Dai)

Supplementary Table S3: List of siRNAs used in this study.

oligonucleotides	Sequence	Reference
AllStars Negative Control siRNA	Proprietary	Qiagen,Venlo,Netherlands
negative control siRNA (targeting firefly luciferase)	5’- CGUACGCGGAAUACUUCGATT-3’	[3]
siRNA against EXOSC10 #1	5’-GAAGGCAGCUGAGCAAACATT-3’	[1]
siRNA against EXOSC10 #2	5’- UGAGCAGAGUAAUGCAGUATT-3’	[1]
siRNA against EXOSC10 #3	5’- AGAUGAAAGUUACGGAUAUTT -3’	[1]
siRNA against SENP3	5’- CAAUAAGGAGCUACUGCUATT -3’	[4]
siRNA against USP36 Hs_USP36_3 (SI00138082)	5’-CAAGAGCGTCTCGGACACCTA-3’	Qiagen,Venlo,Netherlands
siRNA against p14 ARF Hs_CDKN2A_15 (SI02664403)	5’-TACCGTAAATGTCCATTTATA-3’	Qiagen,Venlo,Netherlands
siRNA against HIF-1α	5’-AGGAAGAACTATGAACATAAA-3’	Qiagen,Venlo,Netherlands
siRNA against HIF-2α	5’-CCCGGATAGACTTATTGCCAA -3’	Qiagen,Venlo,Netherlands

Supplementary Table S4: List of the antibodies used in immunoblotting and immunofluorescence experiments.

Antibody/Cat.number	Working dilution	Reference/Source
rabbit polyclonal anti-EXOSC10 (ab50558)	WB: 1/2500, IF: 1/500	Abcam
mouse monoclonal anti- EXOSC10 (B-8: sc-374595)	WB: 1/1000, IF: 1/250	Santa Cruz Biotechnology (Texas,USA)
mouse monoclonal anti-UBF (F-9:SC-13125)	WB: 1/1000, IF: 1/500	Santa Cruz Biotechnology (Texas,USA)
rabbit polyclonal anti-DIS3 (14689-1-AP)	WB: 1/2000	Proteintech
rabbit polyclonal anti- SKIV2L2 (12719-2-AP)	WB: 1/5000	Proteintech
rabbit polyclonal anti-MPHOSPH6 (10695-1-AP)	WB: 1/2000	Proteintech
rabbit polyclonal anti-C1D (10711-1-AP)	WB: 1/1000	Proteintech
rabbit polyclonal anti-EXOSC3 (15062-1-AP)	WB: 1/2000	Proteintech
rabbit polyclonal anti-USP36 (14783-1-AP)	WB: 1/1000, IF: 1/250	Proteintech
mouse monoclonal anti-USP36 (68165-1-Ig)	WB: 1/1000	Proteintech
mouse monoclonal anti-EXOSC2 (66099-1-Ig)	WB: 1/2000	Proteintech
rabbit polyclonal anti-Phospho-p70 S6 Kinase (Thr389) (108D2)	WB: 1/1000	Cell Signaling (Massachusetts, USA)
rabbit polyclonal anti-p14 ARF (E3X6D)	WB: 1/500	Cell Signaling (Massachusetts, USA)
rabbit polyclonal anti- Fibrillarin (C13C3)	WB: 1/1000, IF:1/250	Cell Signaling (Massachusetts, USA)
rabbit polyclonal anti- SENP3 (D20A10)	WB: 1/3000, IF: 1/500	Cell Signaling (Massachusetts, USA)
rabbit polyclonal anti-HA-tag (C29F4)	WB: 1/5000	Cell Signaling (Massachusetts, USA)
mouse monoclonal anti-β-Tubulin (D3U1W)	WB: 1/10000	Cell Signaling (Massachusetts, USA)
mouse monoclonal anti-NPM1 (MAB937)	WB: 1/1000, IF:1/500	Merck Millipore (Massachusetts, USA)

affinity purified rabbit polyclonal anti- HIF-1α	WB 1/500, IF:1/200	[5]
mouse monoclonal anti-HIF-1α (54/HIF1a 610959)	WB: 1/5000, IF:1/100	BD Transduction Laboratories (New Jersey, USA)
rabbit polyclonal anti-HIF-2α (NB100-122)	WB: 1/2000	Novus Europe
mouse monoclonal M2 clone anti-Flag-tag (F1804, 1:2000 dilution)	WB: 1/2500, IF:1/500	Sigma-Aldrich (St Louis, MO)
affinity purified polyclonal anti-SUMO-1	WB: 1/1000	Kindly provided by Melchior Lab (ZMBH, Germany)
mouse monoclonal anti-SENP1 (C-12)	WB: 1/1000	Santa Cruz Biotechnology (Texas,USA)
Mouse monoclonal anti-Ubiquitin (F-11)	WB: 1/1000	Santa Cruz Biotechnology (Texas,USA)
Mouse monoclonal phosphoserine anibody (Clone 19/pSer, 612546)	WB: 1/500	BD Transduction Laboratories (New Jersey, USA)

Supplementary Table S5: Sequences of primers used in qPCR for the amplification of the indicated genes.

oligonucleotides	Sequence	Source
CA9 For	5’-TGGCTGCTGGTGACATCCTA-3’	Eurofins
CA9 Rev	5’-TTGGTTCCCCTTCTGTGCTG-3’	Eurofins
SENP2 For	5’- CAGTTCTCAAAGAAGTCAGATG-3’	Sigma-Aldrich (St Louis, MO)
SENP2 Rev	5’-GTTTTGACTCCGTGATTTTG-3’	Sigma-Aldrich (St Louis, MO)
FOLR1 For	5’-GAGGTTCTATGCTGCAGCCA-3’	Eurofins
FOLR1 Rev	5’-CATTAGGGCCAGGCTAAGCA -3’	Eurofins
RPLP1 For	5’- AAGCAGCCGGTGTAAATGTTGAGC -3’	Eurofins
RPLP1 Rev	5’-CATTGCAGATGAGGCTCCCAATGT-3’	Eurofins

Supplementary Figures

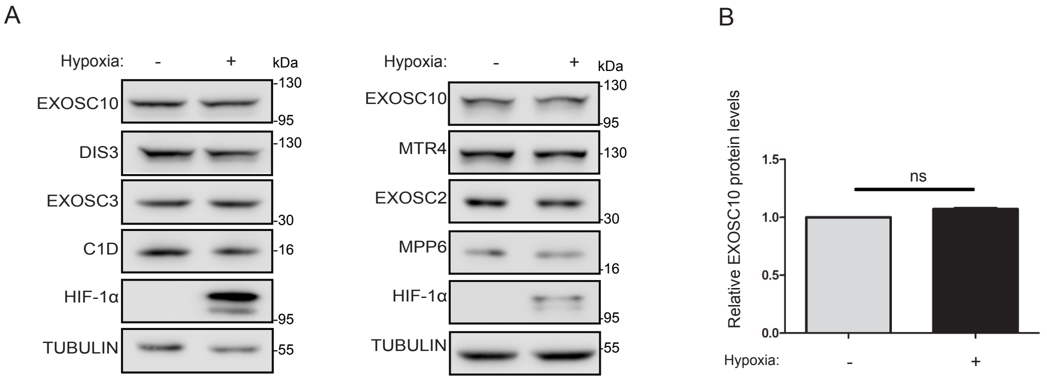

Supplementary Figure S1: Hypoxia does not affect the expression levels of EXOSC10 or other RNA exosome components/cofactors. A. HeLa cells were incubated in normoxia (-) or hypoxia (+) for 24 h and the endogenous expression levels of various RNA exosome components and cofactors were analyzed by immunoblotting using the indicated antibodies. Beta-tubulin was used as a loading control. **B.** Quantification of endogenous EXOSC10 expression levels (normalized to tubulin signal). Values are the mean of three independent experiments and are shown as fold increase compared to normoxic control (grey) and as mean ± standard error. For comparisons between groups unpaired t-test was used (n.s.: not significant).

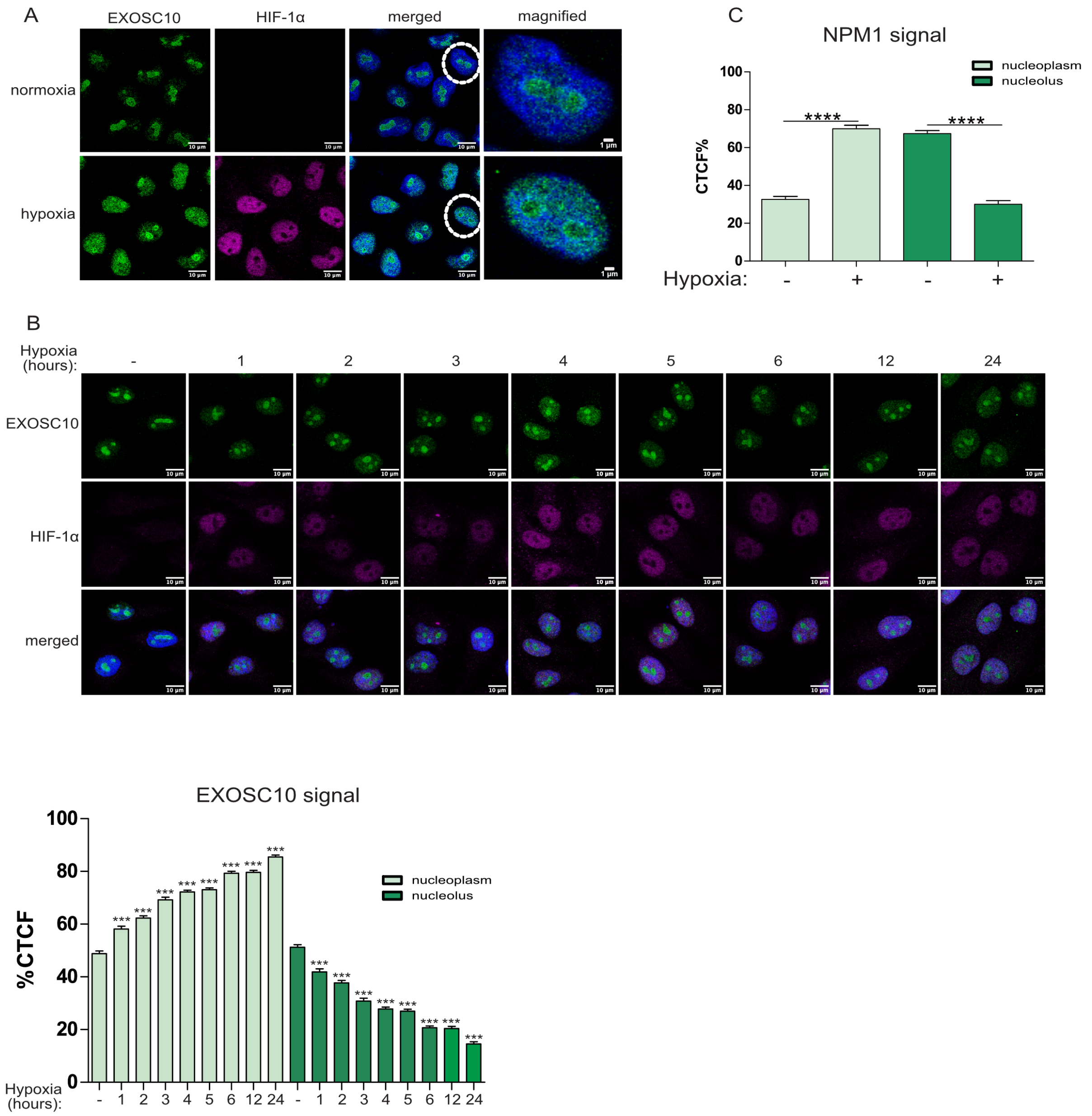

Supplementary Figure S2: Hypoxia-dependent redistribution of EXOSC10 from the nucleolus to the nucleoplasm. A-B. Indirect immunofluorescence analysis of HeLa cells incubated in normoxia or hypoxia for the indicated times, using antibodies against EXOSC10 and HIF-1α (a marker for hypoxic conditions). B (low panel) Graph representing quantification of EXOSC10 IF signal in nucleoli and nucleoplasm from total of 100 cells for each timepoint expressed as percentage of the nuclear cell fluorescence, as mean standard error. For comparisons between two groups ANOVA was used **C**. Graph representing quantification of the NPM1 IF signal (see Fig 2B) (corrected cell fluorescence-CTCF) in, nucleoli and nucleoplasm, from a total of 50 cells for each condition (three independent experiments) expressed as percentage of the nuclear cell fluorescence, as mean ± standard error. For

comparisons between two groups unpaired t-test was used (***: $P<0.001$, n.s.: not significant). DAPI was used for visualization of nuclear material. Scale bar represents 10 µm or 1 µm as indicated.

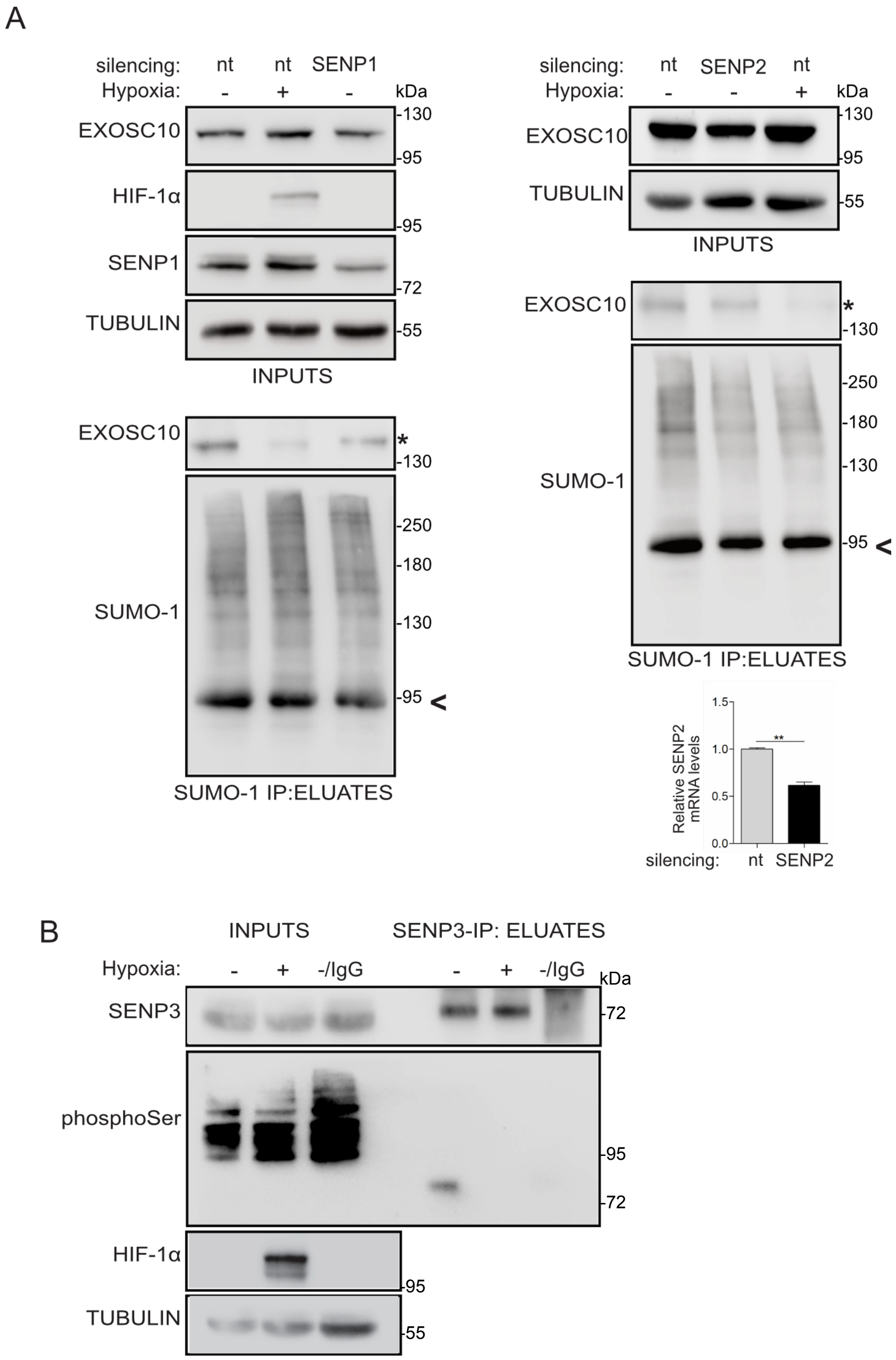

Supplementary Figure S3: A. Silencing of SENP1 or SENP2 does not promote SUMOylation of EXOSC10. HeLa cells were transfected with non-targeted siRNA (nt) or siRNAs against SENP1 (left) or SENP2 (right) and 24 h post-transfection, were incubated in normoxia (-) or hypoxia (+) for 24 h. Cell lysates were subjected to SUMO-1 IP. Inputs and eluates were analyzed by immunoblotting using the indicated antibodies. Silencing of SENP2 was verified with Q-PCR using specific primers for SENP2 mRNA. In all cases, the SUMOylated version of EXOSC10 is indicated with an asterisk (*). SUMOylated RanGAP1

is shown with an arrow and was indicated as a marker for equal loading in INPUTS (A) or as a marker for equal precipitation by anti-SUMO1. **B. SENP3 modulates its phosphorylation status in hypoxia.** HeLa cells incubated in normoxia (-) or hypoxia (+) for 24 h. Cell lysates were subjected to SENP3-IP. An equivalent amount of lysate was also incubated with IgG-beads and served as negative IP control. Soluble extracts (INPUTS) or anti-SENP3 and IgG immunoprecipitates (ELUATE) were analyzed by immunoblotting using the indicated antibodies. Beta-tubulin was used as loading control.

A

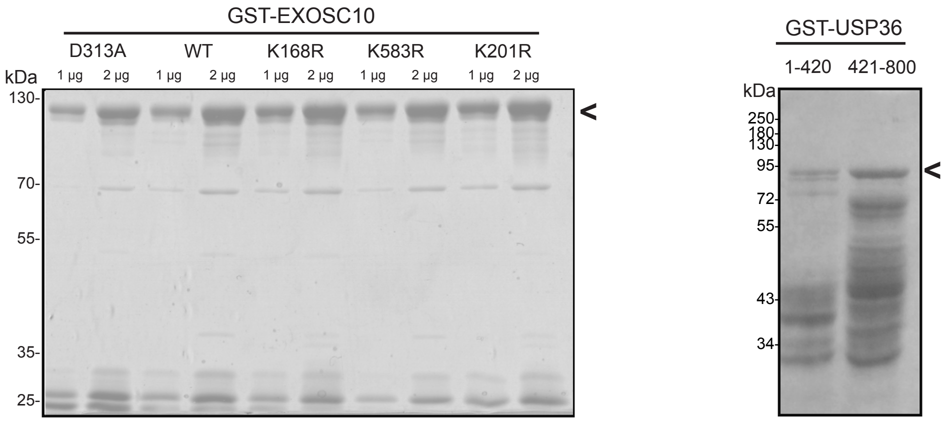

B

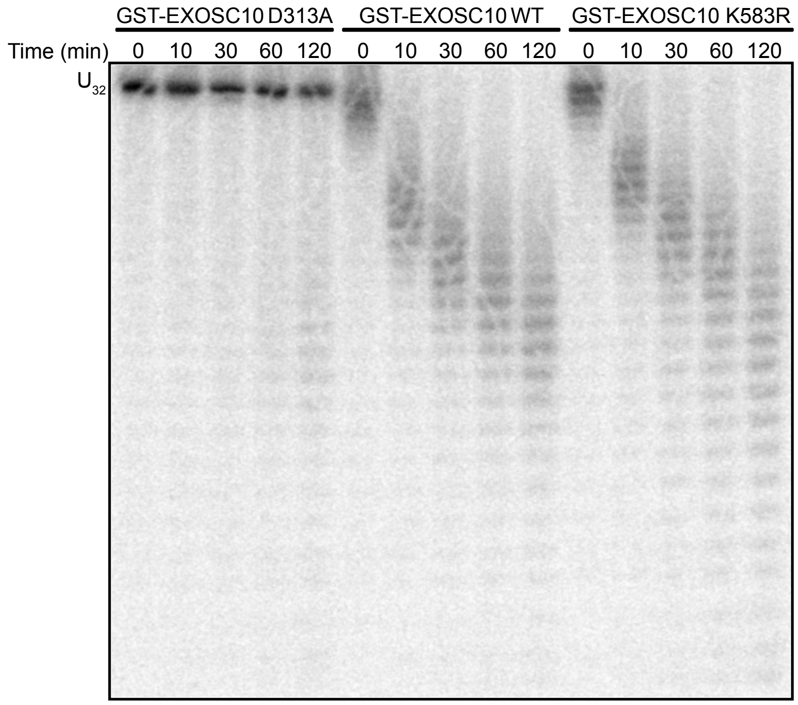

Supplementary Figure S4: Purification and in vitro exoribonuclease assays of recombinant EXOSC10 forms. A. Purified GST-tagged EXOSC10 WT and mutants D313A/K583R/K168R/K201R and GST-USP36 proteins (1-420 and 421-800 forms) were separated by SDS-PAGE and visualized by Coomassie staining. Position of MW is shown on the left. Full length purified proteins are shown with an arrowhead. **B.** *In vitro* exoribonuclease assays were performed using GST-tagged EXOSC10 WT, K583R or catalytically inactive D313A and [^{32}P]-labelled RNA (U32). Samples taken after the indicated times were separated

on a denaturing (8 M urea) 12 % polyacrylamide gel and exposed to a phosphorimager screen before labelled RNAs and RNA fragments were visualized using a phosphorimager.

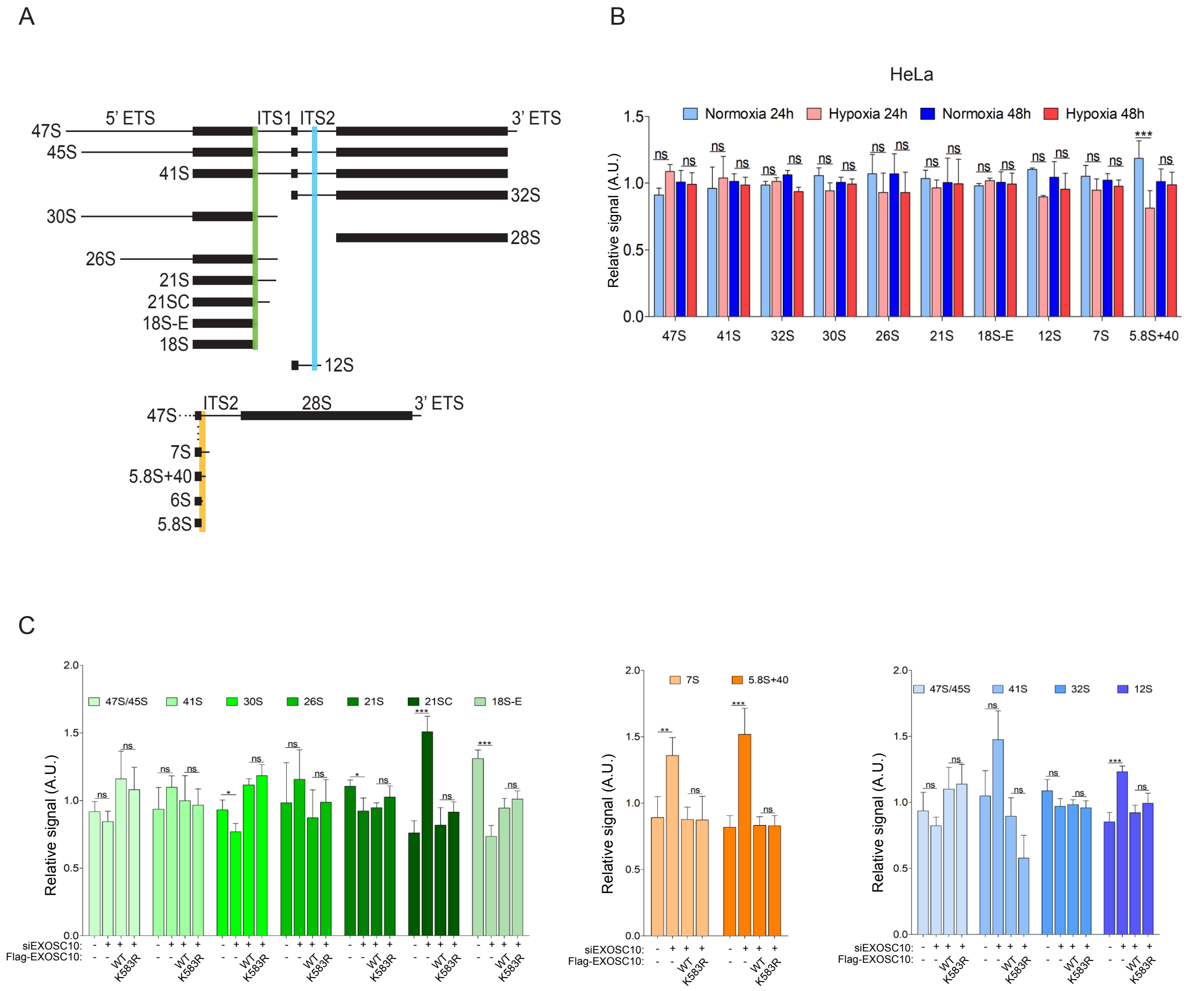

Supplementary Figure S5: Pre-rRNA processing is not affected by deSUMOylation of EXOSC10. A. The major pre-rRNA intermediates present in human cells are depicted schematically. Rectangles depict the mature 18S, 5.8S and 28S rRNAs and lines represent the internal transcribed spacer (ITS1 and ITS2) and the external transcribe spacer (5' ETS and 3' ETS). Colored lines indicate the base-pairing sites of probes used for northern blotting and the pre-rRNA intermediates detected. **B.** Quantification of the northern blot signals from Fig. 6C corresponding to the pre-rRNA intermediates in HeLa exposed to normoxia and hypoxia for 24 h and 48 h. The levels of pre-rRNA intermediates were obtained from three independent experiments, and the signals were quantified relative to the loading controls and shown as mean ± standard deviation. **C.** Total RNA extracted from stably transfected HeLa FlpIn cell lines expressing either the FLAG tag (-), or FLAG-EXOSC10 WT or FLAG-EXOSC10 K583R, in the presence of non-targeted siRNA (-) or a pool of siRNAs against EXOSC10 were analyzed by northern blotting as indicated in Fig. 6D. The levels of pre-rRNA intermediates detected with the probes 3'18S – 5'ITS1 (green), ITS2 (blue), and 3'5.8S – 5'ITS2 (orange) in three

independent experiments were quantified relative to the loading controls and are shown as mean ± standard deviation. For high-molecular-weight pre-rRNAs (green and blue), mitochondrial 16S rRNA was used as loading control. For low-molecular-weight pre-rRNAs (orange), mitochondrial $tRNA^{Phe}$ was used as loading control.

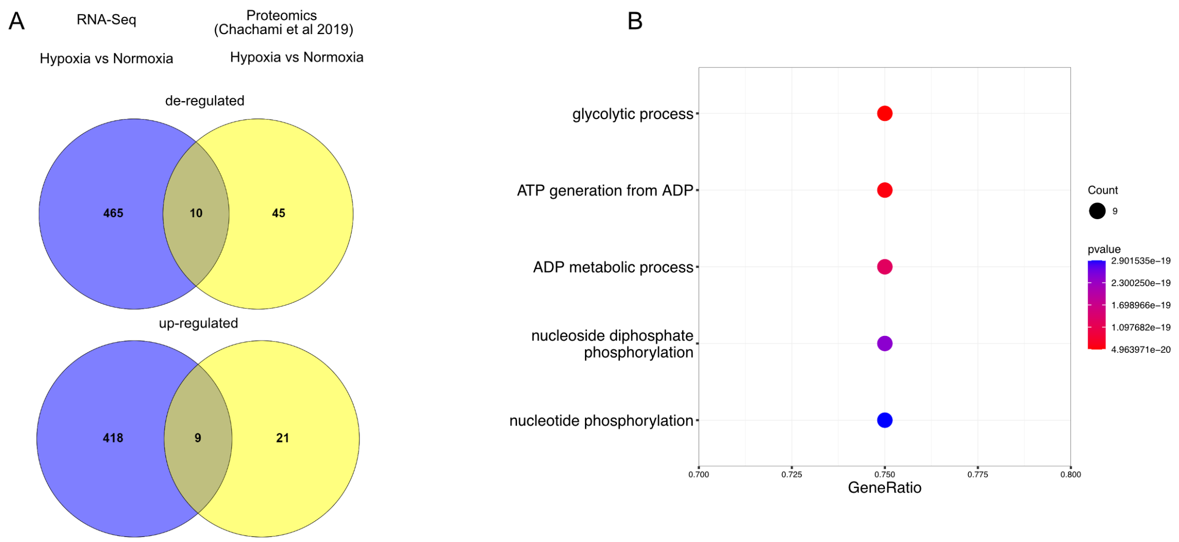

Supplementary Figure S6: A. Upper: Venn diagramm showing overlap between de-regulated genes of hypoxia versus normoxia comparison of RNA-seq data ($\log_2$ FC < -1 | >1, p adj < 0.05) with previously MS data (Chachami et al 2019, log2 FC < -0.5 | >0.5) , down: Venn diagramm showing overlap between up-regulated genes of hypoxia versus normoxia comparison of RNA-seq data (log2 FC > 1, p adj < 0.05) with previously MS (log2 FC > 0.5). **B.** Dotplot highlighting Top5 GO-pathways enriched in HeLa cells using the gene lists generated by comparison of current RNA-seq data (up-regulated genes of hypoxia versus normoxia comparison, log2 FC >1, p adj < 0.05) with previously published MS (Chachami et al 2019) , up-regulated targets of hypoxia versus normoxia comparison, log2 FC >0.5).

References

1. Sloan, K.E., et al., *Both endonucleolytic and exonucleolytic cleavage mediate ITS1 removal during human ribosomal RNA processing.* J Cell Biol, 2013. **200**(5): p. 577-88.
2. Tafforeau, L., et al., *The complexity of human ribosome biogenesis revealed by systematic nucleolar screening of Pre-rRNA processing factors.* Mol Cell, 2013. **51**(4): p. 539-51.
3. Elbashir, S.M., et al., *Duplexes of 21-nucleotide RNAs mediate RNA interference in cultured mammalian cells.* Nature, 2001. **411**(6836): p. 494-8.
4. Huang, C., et al., *SENP3 is responsible for HIF-1 transactivation under mild oxidative stress via p300 de-SUMOylation.* EMBO J, 2009. **28**(18): p. 2748-62.

5. Lyberopoulou, A., et al., *MgcRacGAP interacts with HIF-1alpha and regulates its transcriptional activity.* Cell Physiol Biochem, 2007. **20**(6): p. 995-1006.
6. Chen, Y., et al., *The ubiquitin-specific protease USP36 SUMOylates EXOSC10 and promotes the nucleolar RNA exosome function in rRNA processing*. Nucleic Acids Res, 2023.
